# Management and documentation of pneumonia – a comparison of patients consulting primary care and emergency care

**DOI:** 10.1080/02813432.2024.2326469

**Published:** 2024-03-09

**Authors:** Louise Arntsberg, Sara Fernberg, Ann-Sofie Berger, Katarina Hedin, Anna Moberg

**Affiliations:** a Hälsan 2 Primary Health Care Centre, Jönköping, Sweden; b Åby Primary Health Care Centre, Åby, Sweden; c Futurum, Jönköping, Sweden; d Department of Health, Medicine and Caring Sciences, Linköping University, Linköping, Sweden; e Department of Clinical Sciences in Malmö, Family Medicine, Lund University, Malmö, Sweden; f Kärna Primary Health Care Centre, Linköping, Sweden

**Keywords:** Pneumonia, primary care, emergency care, vital signs, severity scoring

## Abstract

Patients may attend either primary or emergency care without referral in Sweden. Guidelines recommend a severity assessment, including assessment of vital signs, to be performed for all patients presenting with suspected pneumonia.

**Objective:**

To compare management and documentation of vital signs, symptoms and infection severity in pneumonia patients seeking primary care and emergency care without referral.

**Design:**

Medical record review of vital signs, examination findings and severity of pneumonia.

**Setting:**

Primary and emergency care.

**Subjects:**

Two hundred and forty patients diagnosed with pneumonia.

**Main outcome measures:**

Vital signs, examination findings and severity of pneumonia. Assessments of pneumonia severity according to the reviewers, the traffic light score and CRB-65.

**Results:**

Respiratory rate, blood pressure, heart rate and oxygen saturation were less often documented in primary care (*p* < .001). Chest X-ray was performed in 5% of primary care patients vs. 88% of emergency care patients (*p* < .01). Primary care patients had longer symptom duration, higher oxygen saturation and lower respiratory rate. In total, the reviewers assessed 63% of all pneumonias as mild and 9% as severe. The traffic light scoring model identified 11 patients (9%) in primary care and 53 patients (44%) in emergency care at high risk of severe infection.

**Conclusions:**

Vital signs were documented less often in primary care than in emergency care. Patients in primary care appear to have a less severe pneumonia, indicating attendance to the correct care level. The traffic light scoring model identified more patients at risk of severe infection than CRB-65, where the parameters were documented to a limited extent.

## Key Points

- Pneumonia patients attending primary care have less affected vital signs than those attending emergency care.
- Vital signs were less documented in primary care than in emergency care.
- Patients with pneumonia seem to attend the correct level of care when they have the possibility to choose without a referral.
- CRB-65 was not possible to count in most primary care patients due to lack of documentation.

## Introduction

Suspected pneumonia is a common reason for seeking healthcare and community acquired pneumonia (CAP) is a leading cause of mortality and morbidity worldwide [1]. Pneumonia symptoms include cough, dyspnea, increased sputum production and general symptoms of infection such as fever, fatigue and muscle pain. The gold standard for pneumonia diagnosis is chest X-ray (CXR) [2,3]. Since CXR is usually not available at primary health care centers (PHCCs), the diagnosis is often based solely on clinical findings. C-reactive protein (CRP) is recommended in case of uncertainty after assessment [4]. Consequently, the determination of the presence or absence of pneumonia becomes subjective. The use of CXR and CRP differs between countries where CRP is widely used in Scandinavia, whereas CXR is more often used in Spain [5]. We also know that General practitioners (GPs) prescribe antibiotics despite a negative CXR in every other patient [6]. The recommended first line treatment differ between countries [2, 4, 7]. In Sweden, the first-line oral treatment for CAP is phenoxymethylpenicillin. If the patient is allergic to phenoxymethylpenicillin and in case of treatment failure, the recommendation is to treat the patient with doxycycline [4].

When pneumonia is suspected, it is also suggested to assess the severity of the infection, which includes examination of several vital signs [8]. One tool for severity assessment recommended in Swedish primary care is a modified version of the traffic light score based on NICE guidelines for children and the NEWS scoring system [9,10]. It is intended to assist the primary care physician in identifying patients at risk of severe infection [11]. Former studies have suggested that GPs measure and document vital signs only in a minority of patients consulting with an acute infection [12–14].

Worldwide, several clinical tools are used to estimate the severity of an infection, including the Pneumonia Severity Index (PSI) and CRB-65 or CURB-65. CRB-65 may be used in both emergency and primary care and includes the clinical parameters; age, respiratory rate, any confusion and blood pressure. CURB-65 also includes blood samples and is therefore not suitable for primary care [15,16]. The Swedish Association of Infectious Diseases recommends physicians to use CRB-65 to estimate the risk for 30-day mortality. However, CRB-65 may overestimate the risk for severe CAP and therefore should be used with caution [17].

In Sweden, it is possible for patients to attend either primary care or emergency care directly without a referral. In most Swedish Emergency Departments (EDs), patients undergo triage enabling a more systematic documentation of vital signs [18]. Also, EDs have greater access to certain laboratory tests and radiology services. Consequently, the assessment of a patient may depend on diverse information across different care settings.

It is not known if patients seeking medical care for suspected pneumonia, without a referral, differ in severity in primary care vs. emergency care. Furthermore, it is unclear how frequently vital signs and clinical symptoms are recorded in the electronic health records (EHRs) when patients seek medical care for suspected pneumonia.

Our hypothesis was that vital signs were less often documented in primary care compared to emergency care. The aim of this study was to compare management and documentation of vital signs and symptoms as well as the severity of the pneumonia in patients consulting primary care and emergency care.

## Materials and methods

This study conducts a retrospective chart review of EHRs for patients diagnosed with pneumonia (J10–J18) during 2019. The study includes patients 18 years and older diagnosed with pneumonia in publicly run PHCCs and EDs in two regions in south-eastern Sweden. A total of 120 patients were included from primary care and 120 patients from emergency care. Patients were excluded if diagnosed with pneumonia within six weeks before the current visit, if referred from primary care to emergency care, or if the visit took place between 17:00 and 08:00.

### Data collection

**Figure 1.**
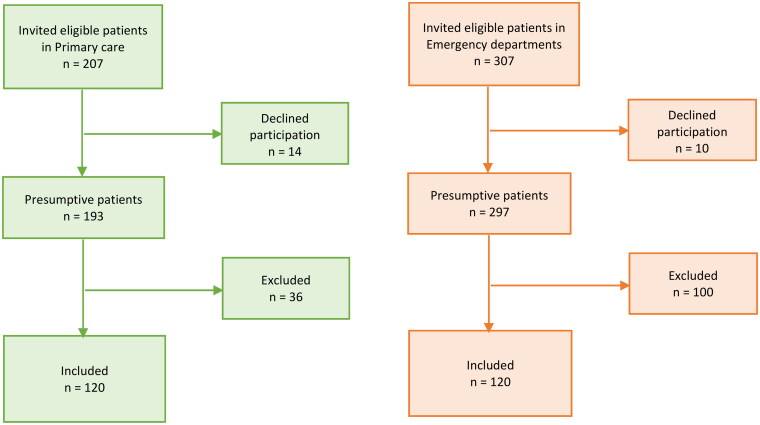
Flowchart of the inclusion process.

Patients were identified through databases in two counties. The first 6–8 identified patients each month during 2019 from primary care and emergency care received written information about the study (*n* = 207 vs. 307) and were given an option to decline participation. Consecutive screening of EHRs was performed until 4–6 patients each month, in each region meeting the inclusion criteria, were identified, until 120 participants in each group were included (Figure 1). For patients who fulfilled the inclusion criteria, a review of the EHRs was made through a study-specific template that included the following data: age, gender, smoking, heart rate, allergy to penicillin, CXR, blood pressure, co-morbidities, respiratory rate, general condition, body temperature, oxygen saturation, antibiotic treatment, days with symptoms, level of consciousness, auscultations of lungs, CRP, white blood cell (WBC) count and immunosuppressive treatment. Co-morbidities included were diabetes, malignancy, lung disease, kidney failure, neurological disease and cardiovascular disease. During the journal review, the reviewers subjectively classified the severity of the pneumonia as mild, moderate or severe. Thereafter, the collected data were pseudonymized and transferred to a database. Based on results from the examinations, patients were scored according to the traffic light system and CRB-65. The traffic light score classifies patients into three categories: green (low risk for severe infection), yellow (intermediate risk) and red (high risk).

### Statistical methods

Demographics are presented as numbers, proportions and medians. Differences in proportions between two independent groups were compared using Pearson’s Chi-squared test. Mann–Whitney’s *U*-test was performed for skewed data. Since the main aim was to compare the actual populations, no adjustments for age and comorbidities were done. The significance level was set to .05 for all analyses and the confidence intervals to 95%. The analyses were performed using IBM SPSS statistics version 27 (Armonk, NY).

The sample size calculation in this descriptive study is based on previous research. To ensure meaningful comparisons, each patient represents at least 1% of the study population, necessitating a minimum of 100 patients per group. Additionally, an allowance for anticipated attrition led to the inclusion of an extra 20 participants per group, enhancing the study’s robustness.

## Results

### Characteristics

There was no significant difference in age between primary and emergency care patients. Gender distribution differed between the groups and comorbidities were less common in patients seeking primary care. The most prevalent comorbidity was cardiovascular disease. In primary care, most patients were assessed by a specialized physician (a specialist in general practice) and in emergency care, most patients were assessed by a junior physician or a resident physician in internal medicine (Table 1).

**Table 1.** Characteristics of adult persons diagnosed with pneumonia in primary care and emergency care.

	Primary care, *n* = 120	Emergency care, *n* = 120	*p* Value
Gender, women, *n* (%)	75 (63)	58 (48)	**.027**
Age, median (range)	61 (18-91)	73 (26-98)	.198
Comorbidity, *n* (%)	69 (58)	101 (84)	**<.001**
Cardiovascular, *n* (%)	36 (30)	74 (62)	**<.001**
COPD, *n* (%)	10 (8)	18 (15)	**<.001**
Asthma, *n* (%)	27 (23)	14 (12)	**<.001**
Level of competence			
Junior physician^a^, *n* (%)	31 (26)	27 (23)	**<.001**
Resident and licensed medical physicians, *n* (%)	34 (28)	76 (63)	**<.001**
Specialized physician, *n* (%)	55 (46)	17 (14)	**.003**

^a^ Early in the career, before license.P-values < .05 were considered as significant and are bolded in the table.

### Documentation

The frequency of documenting clinical findings in the EHRs varied between primary care and emergency care. In primary care, the reviewed vital signs were documented in 24–75% of the patients vs. in 83–88% of the emergency care patients (Table 2). In total, all vital signs were documented in 14% in primary care vs. 74% in emergency care (*p* < .001). Significant differences were found for the documentation of heart rate, blood pressure, respiratory rate, oxygen saturation and the number of CRP and WBC tests (Table 2). Smoking habits were documented only to a limited extent in both primary care and emergency care.

**Table 2.** Documentation of clinical findings in adults diagnosed with pneumonia in primary care and emergency care.

	Primary care, *n* = 120, *n* (%)	Emergency care, *n* = 120, *n* (%)	*p* Value
Duration of symptoms	111 (93)	105 (88)	.197
Smoking	30 (25)	27 (23)	.65
General condition	116 (97)	108 (90)	**.038**
Consciousness	56 (47)	63 (53)	.366
Respiratory rate	29 (24)	100 (83)	**<.001**
Oxygen saturation	84 (70)	106 (88)	**<.001**
Blood pressure	40 (33)	104 (87)	**<.001**
Heart rate	77 (64)	102 (85)	**<.001**
Body temperature	90 (75)	99 (83)	.156
Skin	3 (2.5)	8 (6.7)	.123
Urine production	6 (5)	21 (18)	**.002**
Auscultation of lungs	119 (99)	118 (98)	.561
CRP analyzed	112 (93)	119 (99)	**.017**
WBC analyzed	37 (31)	117 (98)	**<.001**
Referred for CXR	6 (5)	105 (88)	<.001

P-values < .05 were considered as significant and are bolded in the table.

### Clinical findings and actions taken at the examination

Patients with pneumonia diagnosed in primary care in general had a longer duration of symptoms compared to patients seeking emergency care (Table 3). In primary care, 5% were referred for CXR; in emergency care, 88% were examined with CXR. In approximately half of the visits, the physician documented that the patients received information on how and when to seek medical advice in case of unresolved symptoms; there was no difference between primary care (55%) and emergency care (56%).

**Table 3.** Clinical findings in adult patients diagnosed with pneumonia in primary care and emergency care.

	Primary care, *n* = 120	Emergency care, *n* = 120	*p* Value
Duration of symptoms, days	7 (1–42)	3 (1–27)	**<.001**
Smoker, *n* (%)	4 (13)	10 (37)	**.006**
Affected general condition, *n* (%)	35 (30)	48 (44)	**.027**
Lung sound abnormality, *n* (%)	90 (76)	92 (78)	.67
Respiratory rate, *n*/min	19 (14–36)	22 (10–50)	**.016**
Oxygen saturation, %	96 (86–100)	94 (65–100)	**<.001**
Heart rate, /min	86 (59–136)	94 (57–150)	.142
Body temperature, °C	37.5 (36.2–40.0)	38 (34.6–41.0)	.109
Systolic blood pressure, mmHg	127 (106–180)	130 (80–220)	.865
WBC, ×10^9^/L	11.5 (3.2–20.7)	10.5 (1.7–41.1)	.351
CRP, mg/L	78 (4–201)	85 (4–538)	.715
Gender, women, *n* (%)	75 (63)	58 (48)	**.027**
Age, years	61 (18–91)	73 (26–98)	0.198

Data are presented as medians and (range) unless otherwise stated. P-values < .05 were considered as significant and are bolded in the table.

### Severity assessment by the reviewers

**Figure 2.**
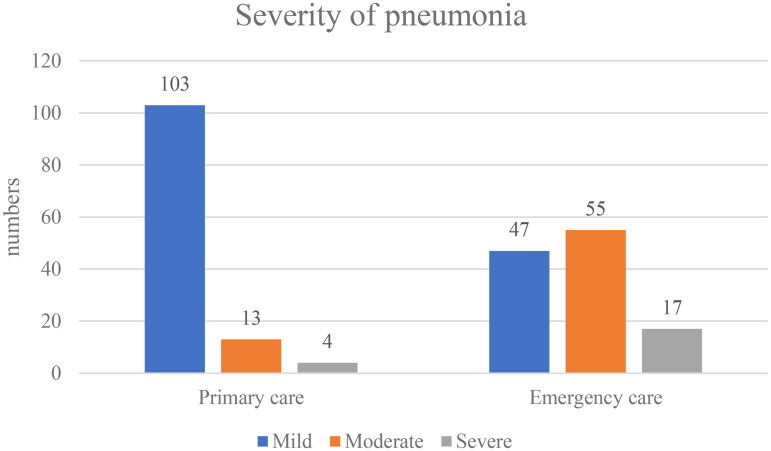
Severity of pneumonia diagnosed in primary care and emergency care assessed by the physicians reviewing the medical records.

When comparing the reviewers’ assessment of patients in primary care with those in emergency care, there was a difference in the severity of the infection where 86% of the primary care patients were considered as having a mild pneumonia vs. 39% of the emergency care patients (Figure 2). Of the 17 patients in primary care who were scored as having a moderate or severe infection, eight (70%) were referred to hospital and six of these were hospitalized. Patients attending emergency care were more often scored as having moderate or severe infection, and 79 patients of 119 (66%) were hospitalized. Of the 79, 11 were considered mild, 50 moderate and 17 severe. In one case, the classification of the severity was indeterminable.

### Scoring with traffic light score and CRB-65

**Figure 3.**
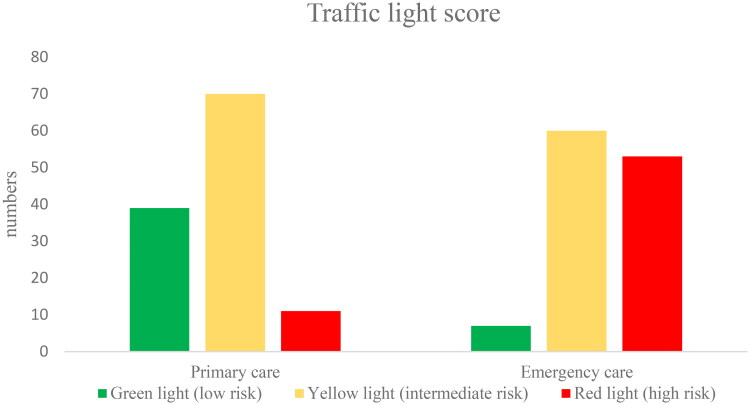
Severity of pneumonia in adult patients according to the traffic light score.

When scoring the risk of severe illness using the traffic light scoring, 11 patients in primary care were scored red (Figure 3). Of these, five were referred to hospital for further management. The remaining patients were treated in primary care.

**Figure 4.**
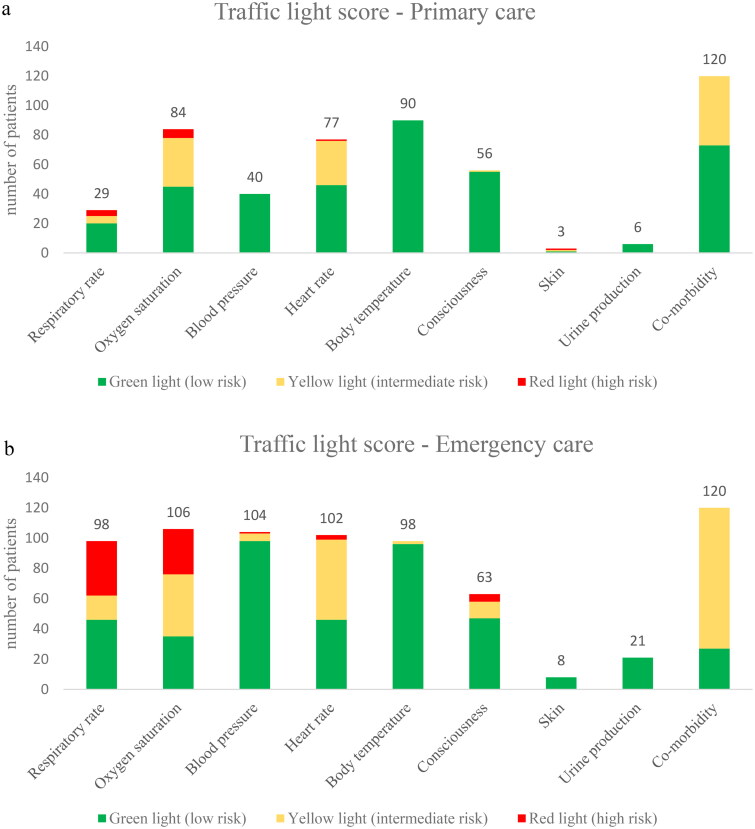
(a, b) Number of patients in each category according to the traffic light score.

Most patients scoring red had elevated respiratory rate and lowered oxygen saturation (Figure 4(a,b)). When estimating CRB-65, all four components – new onset confusion, respiratory rate (≥30/min), blood pressure (<90 mmHg systolic or ≤60 mmHg diastolic), age ≥65 years – were documented in 7.5% of primary care patients and in 45% of emergency care patients.

### Antibiotics

Antibiotics were prescribed to 114 (95%) patients in primary care vs. 117 (98%) in emergency care. When oral treatment was prescribed, phenoxymethylpenicillin was used more frequently in primary care (61%) than in emergency care (41%) (*p* < .001). Doxycycline was prescribed in 29% of patients in primary care vs. 24% in emergency care. A documented penicillin allergy was identified in nine patients (27%) assessed in primary care and one patient (10%) assessed in emergency care.

## Discussion

### Summary of main findings

This retrospective study reveals that all vital signs needed for severity assessment according to the traffic light score were documented in 14% of patients diagnosed with pneumonia in primary care vs. 74% in emergency care. In primary care, less than one out of 10 patients with pneumonia scored as having a high risk of severe infection. Patients in emergency care more often presented with deviant parameters, mainly higher respiratory rate, and lower oxygen saturation than patients in primary care. Barely every other patient scored as having a high risk of severe infection. When comparing the outcome of the different scores, assuming that lack of documentation corresponded to normal findings, the traffic light score and the severity assessed by the reviewers resulted in higher severity than CRB-65 in both groups.

### Strengths and limitations

A strength of this study is that patients were only included if they attended EDs during the opening hours of the primary care (8:00–17:00) in order to exclude those who were seeking emergency care during evenings and nights with mild symptoms of pneumonia who, under normal circumstances would have sought primary care during the daytime. Another strength is that all patients diagnosed with pneumonia within six weeks before the index visit were excluded to ensure a newly onset of the disease. The retrospective design is also suitable since the results reflect the actual clinical work in primary care and emergency care. However, the design only allowed us to review what was documented and not what was actually examined, which is a limitation. Another limitation is that only publicly run PHCCs were available, which might have allowed inclusion of patients who had consulted private care during the six weeks before the index visit. We identified patients who were diagnosed with pneumonia, but we could not be certain that they did not have for example acute bronchitis or acute exacerbation of COPD. Moreover, assessments in primary and emergency care rely on diverse information, given disparities in access to different examinations. It is also possible that the physicians assigned the pneumonia diagnosis to justify prescribing antibiotics. This is of course a limitation. Furthermore, a potential limitation of the study is the lack of information regarding patient behavior and expectations which could have had impact on the health care provider.

### Comparison with existing literature

The poor documentation of vital signs in primary care is in line with a study by Francis et al. [14], who showed that physicians in primary care only documented respiratory rate in 23% and blood pressure in 32%. In emergency care, a majority of vital signs were documented. The most notable differences between primary care and emergency care were for respiratory rate (24% vs. 83%) and blood pressure (33% vs. 87%). There are several possible explanations for the differences in documentation in primary care and emergency care. In emergency care, all patients undergo triage by a nurse enabling systematic documentation of vital signs. In addition, patients in emergency care are generally at risk of more severe pneumonia, which reinforce the need to monitor vital signs. The documentation of vital signs is currently insufficient in primary care. There is a need for improvement, not only to enable estimation of severity of pneumonia but also to enhance assessment capabilities during follow-up sessions. Increasing the documentation of vital signs may be crucial, yet the correlation between enhanced documentation and improved patient care remains uncertain. While it is likely that such efforts may capture additional patients at high risk of severe infection and possibly contribute to a reduction in the duration of hospital stays for some individuals, the fundamental question of whether these practices genuinely lead to better patient outcomes remains unanswered.

It was quite surprising that the use of CXR differed so much (5% vs. 88%). The low use of CXR in primary care contrasts with previous studies indicating a usage of 12–22% in cases of suspected pneumonia; however, the methodologies and study populations differ [19,20]. The finding suggests that emergency care routinely uses CXR when pneumonia is suspected. In primary care, CRP was analyzed in 93% vs. 99% in emergency care. This finding is not surprising as blood samples are routinely collected before patients are examined by an emergency care physician. The excessive use of CRP when diagnosing pneumonia in primary care has been shown before [19, 21].

Although patients in emergency care presented with more deviant vital signs than patients in primary care, the difference in the severity of the pneumonia was not extreme. According to the reviewer’s assessment, patients attending primary care in most cases had a mild or moderate risk of severe pneumonia but rarely a high risk. This finding suggests that patients who consult primary care diagnosed with pneumonia generally attend the correct level of care. The patients in emergency care had a shorter duration of symptoms, lower oxygen saturation and higher respiratory rate, which indicates a more severe infection. The primary care population consisted of more women and was slightly (but not significant) younger, than the emergency care patients. Moreover, patients from emergency care presented with more comorbidities. Since we aimed to calculate severity and compare the groups as in real life, we chose not to adjust for these differences in our analyses of examination findings, thus results from vital signs can be biased. The fact that more women were diagnosed with pneumonia in primary care in this study is interesting and might indicate a need of further research. However, the gender distribution in primary care is in line with the study by Christensen et al. where the proportion of women with pneumonia was 60% [5]. In hospital, the proportion has been shown to be the inverse [22].

Assuming mild pneumonia corresponds to low risk, moderate pneumonia to intermediate risk, and severe pneumonia to high risk of severe infection, there were differences between the scoring systems. According to the reviewers, 13 primary care patients were assessed as having moderate pneumonia compared to the traffic light score, where 70 patients were identified as having an intermediate risk of severe infection. Co-morbidity emerged as a parameter that might explain this difference. In the traffic light score, co-morbidity is classified as ‘yes’ or ‘no’; however, when reviewing EHRs, a comprehensive assessment might result in a lower score.

When calculating CRB-65 scores, all four components are needed. In the current study, CRB-65 could only be estimated in 7.5% of the patients in primary care and 45% of the patients in emergency care. This difference could be explained by the fact that only diverging findings have been documented. This explanation was also provided in an observation study by Launders et al. [23], where level of confusion was sparsely documented which might reflect the absence of recording a normal mental status.

Nearly everyone in this study received antibiotics, however, a few lacked documentation regarding prescription. When looking for the reason for this, it appears that they had severe infections leading to hospitalization, suggesting that information regarding antibiotic prescription was unavailable to the reviewers. In primary care, first-line antibiotics were predominantly prescribed; in emergency care, only two out of five were prescribed first line antibiotics. The vast majority of second line treatment could not be explained by penicillin allergy. Although this is not in line with the recommendations, it is an improvement compared to what Moberg et al. found in a study based on data from 2008 to 2014 [19], suggesting that the prescribing of antibiotics is moving in the recommended direction, at least in primary care.

## Conclusions

Vital signs are less often documented in primary care than in emergency care. Moreover, CXR and CRP are less often used in the assessment of patients with suspected pneumonia, while first-line antibiotics are more often used in primary care. Patients who can choose to seek primary care or emergency care without a referral appear to exhibit more severe infections when choosing emergency care, indicating an alignment with the appropriate level of care. In this study, the traffic light scoring model identified more patients at risk of severe infection and potential need for hospital care than CRB-65, where the parameters were documented to a limited extent, particularly in primary care. Future studies are needed to validate the traffic light model for its applicability in pneumonia patients in primary care.

## Data Availability

The datasets generated and analyzed during the current study are not publicly available due to Swedish legislation (the Personal Data Act) but are available from the corresponding author on reasonable request.
